# Supplementary material for: Don’t worry, be active: how to facilitate the detection of errors in immersive virtual environments
Source: PeerJ. 2018 Oct 29;6:e5844. doi: 10.7717/peerj.5844 (PMC6211266; doi:10.7717/peerj.5844)
Supplement: Supplemental Information 2 — Sources of information accessible to active and passive observers in standard IVR systems. [file peerj-06-5844-s002.doc]

## Supplemental text

## Sources of information accessible to active and passive observers in standard IVR systems

Our main expectation of a possible active *vs*. multi-passive viewing advantage in standard collaborative IVR systems is based on three major sources of available information to active and passive observers:

1. *Stereoscopic (in active)* *vs. monoscopic cyclopean (in passive) view*.

The active observer is exposed to a self-generated stereoscopic immersive view of the layout, whereas the passive observer receives a monoscopic cyclopean immersive view of the exact-same layout. The stereoscopic vision is known to generate a relative advantage in the spatial reconstruction of 3D structures (Hay, 1966; Koenderink & van Doorn, 1975; Koenderink & van Doorn, 1976; Ullman, 1979; Longuet-Higgins, 1984; Ullman, 1986; Rogers & Bradshaw, 1995; Barfield, Hendrix, & Bystrom, 1997; Fantoni, 2008), and spatial layout (e.g., Arms, Cook, & Cruz-Neira, 1999; Ruddle, Pyne & Jones, 1999; Raja et al., 2004; Paes, Arantes & Irizarry, 2017) over the monoscopic vision. This advantage has been shown to support spatial judgments which are typically required for precise visual inspections and for comparisons that are typical of collaborative design review sessions (Gruchalla, 2004; Forsberg et al., 2008; Ragan et al., 2013).

1. *Presence (in active) vs. absence (in passive) of Idiothetic information, agency and intentionality*.

Active exploration, but not passive observation, involves two peculiar types of activities: (1) physical activity due to motor control involving reafferent proprioceptive and vestibular information from sensed ego-motion; i.e., idiothetic information; (2) and cognitive activity due to attention, decision making and mental manipulation, involving agency and intentionality (Wilson et al., 1997; Chrastil & Warren, 2012). Accumulated evidence showed that these differential activities impact on 3D viewing, spatial learning and object recognition. As regards idiothetic information, theoretical models on self-generated (not passively observed) dynamic visual layouts have shown that, in principle, a veridical reconstruction of the 3D shape and motion of environmental objects can be achieved if the first-order optic information is optimally combined with extraretinal signals (Sherrington, 1906; von Helmholtz, 2002; Colas et al., 2007; Caudek, Fantoni & Domini, 2011). In line with these models, active 3D viewing has been shown to disambiguate several 3D properties of environmental objects (i.e., shape, depth, planar surface slant, rigidity and motion), through the optimal integration of retinal and extra-retinal information regarding ego-motion (Ono & Steinbach, 1990; Ujike & Ono, 2001; Peh et al., 2002; Naji & Freeman, 2004; Ono & Ujike, 2005; Wexler & van Boxtel, 2005; Fantoni, Caudek & Domini, 2010; Caudek, Fantoni & Domini, 2011; Fantoni, Caudek & Domini, 2012; Fantoni, Caudek & Domini, 2014). Another strand of studies has considered the impact of idiothetic information on spatial learning. In particular, Chrastil and Warren (2012) discussed how active exploration and passive observation with variable fidelity of extraretinal and proprioceptive information might affect spatial navigation and scene/object recognition. The review of the above-mentioned evidence (Chrastil & Warren, 2012) offers a qualified support for an idiothetic contribution to spatial learning, while results are somehow mixed for scene/object recognition (Bülthoff, Mohler & Thornton, 2018; Teramoto & Riecke, 2010). Relative to passive vision alone, the combination of retinal, and extra-retinal information from ego-motion, resulting from the active exploration of a virtual environment with a full body motion tracking system, improved observers’ performance on several types of tasks, such as: pointing (Chance et al., 1998; Waller, Loomis & Haun, 2004), route learning and wayfinding (Riecke et al., 2010; Ruddle, Volkova & Bülthoff, 2011; Ruddle et al., 2011). Such an improvement was found to be directly proportional to the complexity of pathways (Chance et al., 1998; Waller & Greenauer, 2007). As far as object recognition is concerned, mounting evidence has suggested that viewing objects under intentional/active manual control, rather than non-intentional/passive observation, increases detection speed and sensitivity in both 3D (Harman, Humphrey & Goodale, 1999; James et al., 2002), and 2D space (Ichikawa & Masakura, 2006; Scocchia et al., 2009). Meijer & Van der Lubbe (2011) showed that the improved perceptual sensitivity after intentional exploration is due to action itself, rather than to the changes in the allocation of visual attention involved in different types of viewings. However, such an advantage was found to be less pronounced when comparing the recognition of objects under active and passive viewing, when the dynamic visual information includes the same amount of perspective change, regardless of the environmental or observer motion (Teramoto and Riecke, 2010; Wang & Simons, 1999; Experiment 3 in Christou and Bülthoff, 1999). Recent evidence suggests that an active vs. passive advantage occurs with biologically relevant stimuli like faces: the active learning experience of an avatar face leads to a more robust face recognition performance than its passive reply (Bülthoff, Mohler, & Thornton, 2018; Liu, Ward & Markall, 2007).

1. *High (in active) vs. low (in passive) cognitive load*.

Compared to active exploration, any passive observation of a 3D scene comparable in terms of visual input involves a lower mental effort thus implying a lower cognitive load. The passive observation of a dynamic scene does not require the planning of action needed to explore/interact within/with the immersive virtual environment. Furthermore, the passive observation generally involves relatively less demanding manual operation than active exploration. On this basis, Liu, Ward and Markall (2007) hypothesized a reduction of the attentional resources in active exploration relative to passive observation that should result in an active vs. passive disadvantage in object recognition. In partial agreement with this expectation Liu, et al. (2007, Experiment 6 and 7) found that the reduction of attentional resources consistently affects the recognition of biologically irrelevant stimuli, like chairs, but not biologically relevant stimuli, like faces.

## References

Arms L, Cook D, Cruz-Neira C. 1999. The benefits of statistical visualization in an immersive environment. In Proceedings of IEEE Virtual Reality 1999. Huston: IEEE, 88-95. DOI: 10.1109/VR.1999.756938

Bülthoff I, Mohler BJ, Thornton IM. 2018. Face recognition of full-bodied avatars by active observers in a virtual environment. Vision Research. DOI: [10.1016/j.visres.2017.12.001](https://doi.org/10.1016/j.visres.2017.12.001)

Caudek C, Fantoni C, Domini F. 2011. Bayesian modeling of perceived surface slant from actively-generated and passively-observed optic flow. *PloS One* 6:e18731. DOI: [10.1371/journal.pone.0018731](https://doi.org/10.1371/journal.pone.0018731)

Chance SS, Gaunet F, Beall AC, Loomis JM. 1998. Locomotion mode affects the updating of objects encountered during travel: The contribution of vestibular and proprioceptive inputs to path integration. *Presence: Teleoperators and Virtual Environments* 7:168–178. DOI: [10.1162/105474698565659](https://doi.org/10.1162/105474698565659)

Chrastil ER, Warren, WH. 2012. Active and passive contributions to spatial learning. *Psychonomic Bulletin Review* 19:1-23. DOI: 10.3758/s13423-011-0182-x

Christou CG, Bülthoff HH. 1999. View dependence in scene recognition after active learning. *Memory & Cognition* 27:996-1007. DOI: 10.3758/BF03201230

Colas F, Droulez J, Wexler M, Bessiere P. 2007. A unified probabilistic model of the perception of three-dimensional structure from optic flow. *Biological Cybernetics* 97:461–477. DOI: [10.1007/s00422-007-0183-z](https://doi.org/10.1007/s00422-007-0183-z)

Fantoni C. 2008. 3D surface orientation based on a novel representation of the orientation disparity field. *Vision Research* 48:2509-2522. DOI: [10.1016/j.visres.2008.08.015](https://doi.org/10.1016/j.visres.2008.08.015)

Fantoni C, Caudek C, Domini F. 2010. Systematic distortions of perceived planar surface motion in active vision. *Journal of Vision* 10:12. DOI: 10.1167/10.5.12

Fantoni C, Caudek C, Domini F. 2012. Perceived surface slant is systematically biased in the actively-generated optic flow. *PloS One* 7:e33911. DOI: [10.1371/journal.pone.0033911](https://doi.org/10.1371/journal.pone.0033911)

Fantoni C, Caudek C, Domini F. 2014. Misperception of rigidity from actively generated optic flow. *Journal of Vision* 14:10. DOI: 10.1167/14.3.10

Forsberg A, Katzourin M, Wharton K, Slater M. 2008. A comparative study of desktop, fishtank, and cave systems for the exploration of volume rendered confocal data sets. *IEEE Transactions on Visualization and Computer Graphics* 14:551-563. DOI: [10.1109/TVCG.2007.70433](https://doi.org/10.1109/TVCG.2007.70433)

Gruchalla K. 2004. Immersive well-path editing: investigating the added value of immersion. In *Proceedings of IEEE Virtual Reality 2004*. Chicago: IEEE, 157-164. DOI: 10.1109/VR.2004.1310069

Harman KL, Humphrey GK, Goodale MA. 1999. Active manual control of object views facilitates visual recognition. *Current Biology* 9:1315-1318. DOI: [10.1016/S0960-9822(00)80053-6](https://doi.org/10.1016/S0960-9822(00)80053-6)

Hay JC. 1966. Optical motions and space perception – an extension of Gibson’s analysis. *Psychological Review* 73:550-565. DOI: [10.1037/h0023863](http://psycnet.apa.org/doi/10.1037/h0023863)

James KH, Humphrey GK, Vilis T, Corrie B, Baddour R, Goodale MA. 2002. “Active” and “passive” learning of three-dimensional object structure within an immersive virtual reality environment. *Behavior Research Methods, Instruments, & Computers* 34:383-390. DOI: 10.3758/BF03195466

Koenderink JJ, van Doorn AJ. 1975. Invariant properties of the motion parallax field due to the movement of rigid bodies relative to an observer. *Optica Acta* 22:773–791. DOI: [10.1080/713819112](https://doi.org/10.1080/713819112)

Koenderink JJ, van Doorn AJ. 1976. Geometry of binocular vision and a model for stereopsis. *Biological Cybernetics* 21:29-35. DOI: 10.1007/BF00326670

Ichikawa M, Masakura Y. 2006. Manual control of the visual stimulus reduces the flash-lag effect. *Vision Research* 46: 2192-2203.

Liu CH, Ward J, Markall H. 2007. The role of active exploration of 3D face stimuli on recognition memory of facial information. *Journal of Experimental Psychology: Human Perception and Performance* 33:895-904. DOI: [10.1037/0096-1523.33.4.895](http://psycnet.apa.org/doi/10.1037/0096-1523.33.4.895)

Longuet-Higgins HC. 1984. The visual ambiguity of a moving plane. *Proceedings of the Royal Society of London* 223:165–175. DOI: 10.1098/rspb.1984.0088

Meijer F, Van der Lubbe, RH. 2011. Active exploration improves perceptual sensitivity for virtual 3D objects in visual recognition tasks. *Vision Research* 51:2431-2439. DOI: [10.1016/j.visres.2011.09.013](https://doi.org/10.1016/j.visres.2011.09.013)

Naji JJ, Freeman TC. 2004. Perceiving depth order during pursuit eye movement. *Vision Research* 44:3025-3034. DOI: [10.1016/j.visres.2004.07.007](https://doi.org/10.1016/j.visres.2004.07.007)

Ono H, Steinbach MJ. 1990. Monocular without stereopsis with and head movement. *Perception & Psychophysics* 48:179-187. DOI: 10.3758/BF03207085

Ono H, Ujike H. 2005. Motion parallax driven by head movements: Conditions for visual stability, perceived depth, and perceived concomitant motion. *Perception* 34:477-490. DOI: [10.1068/p5221](https://doi.org/10.1068/p5221)

Paes D, Arantes E, Irizarry J. 2017. Immersive environment for improving the understanding of architectural 3D models: Comparing user spatial perception between immersive and traditional virtual reality systems. *Automation in Construction* 84:292-303. DOI: [10.1016/j.autcon.2017.09.016](https://doi.org/10.1016/j.autcon.2017.09.016)

Peh CH, Panerai F, Droulez J, Cornilleau-Pérès V, Cheong LF. 2002. Absolute distance perception during in-depth head movement: Calibrating optic flow with extra-retinal information. *Vision Research* 42:1991-2003. DOI: [10.1016/S0042-6989(02)00120-7](https://doi.org/10.1016/S0042-6989(02)00120-7)

Ragan ED, Kopper R, Schuchardt P, Bowman DA. 2013. Studying the effects of stereo, head tracking, and field of regard on a small-scale spatial judgment task. *IEEE Transactions on Visualization and Computer Graphics* 19:886-896. DOI: [10.1109/TVCG.2012.163](https://doi.org/10.1109/TVCG.2012.163)

Raja D, Bowman DA, Lucas J, North C. 2004. Exploring the benefits of immersion in abstract information visualization. In *Proceedings of Immersive Projection Technology Workshop*. Available at http://people.cs.vt.edu/~bowman/papers/ipt_dheva.pdf (accessed 23 February 2018)

Riecke BE, Bodenheimer B, McNamara TP, Williams B, Peng P, Feuereissen D. 2010. Do we need to walk for effective virtual reality navigation? Physical rotations alone may suffice. In Holscher C, Shipley T, Olivetti Belardinelli M, Bateman J, Newcombe N, eds. *Spatial cognition VII: Lecture notes in computer science*. Berlin: Springer, 234–247. DOI: 10.1007/978-3-642-14749-4_21

Rogers BJ, Bradshaw MF. 1995. Disparity scaling and the perception of frontoparallel surfaces. *Perception* 24:155–179. DOI: [10.1068/p240155](https://doi.org/10.1068%2Fp240155)

Ruddle RA, Payne SJ, Jones DM. 1999. Navigating largescale virtual environments: What differences occur between helmet-mounted and desk-top displays? *Presence: Teleoperators and Virtual Environments* 8:157-168. DOI: [10.1162/105474699566143](https://doi.org/10.1162/105474699566143)

Ruddle RA, Volkova E, Bülthoff HH. 2011. Walking improves your cognitive map in environments that are large-scale and large in extent. *ACM Transactions on Computer–Human Interaction*, 18 [Article No. 10]:1-20. DOI: [10.1145/1970378.1970384](https://doi.org/10.1145/1970378.1970384)

Ruddle RA, Volkova E, Mohler B, Bülthoff HH. 2011. The effect of landmark and body-based sensory information on route knowledge. *Memory & Cognition* 39:686-699. DOI: 10.3758/s13421-010-0054-z

Scocchia L, Grosso RA, de’Sperati C, Stucchi N, Baud-Bovy G. 2009. Observer’s control of the moving stimulus increases the flash-lag effect. *Vision research*, 49: 2363-2370.

Sherrington C. 1906. *The Integrative Action of the Nervous System*. New York: Charles Scribners Sons.

Teramoto W, Riecke BE. 2010. Dynamic visual information facilitates object recognition from novel viewpoints. *Journal of Vision* 10:11. DOI: 10.1167/10.13.11

Ujike H, Ono H. 2001. Depth thresholds of motion parallax as a function of head movement velocity. *Vision Research* 41:2835-2843. DOI: [10.1016/S0042-6989(01)00164-X](https://doi.org/10.1016/S0042-6989(01)00164-X)

Ullman S. 1979. *The interpretation of visual motion*. Cambridge, MA: MIT Press.

Ullman S. 1986. The optical flow of planar surfaces. *Spatial Vision* 1:263–276. DOI: [10.1163/156856886X00070](https://doi.org/10.1163/156856886X00070)

von Helmholtz H. 2002. *Handbook of physiological optics.* New York: Dover.

Waller D, Greenauer N. 2007. The role of body-based sensory information in the acquisition of enduring spatial representations. *Psychological Research* 71:322-332. DOI: 10.1007/s00426-006-0087-x

Waller D, Loomis JM, Haun DB. 2004. Body-based senses enhance knowledge of directions in large-scale environments*. Psychonomic Bulletin & Review* 11:157-163. DOI: 10.3758/BF03206476

Wang RF, Simons DJ. 1999. Active and passive scene recognition across views. *Cognition* 70:191-210. DOI: [10.1016/S0010-0277(99)00012-8](https://doi.org/10.1016/S0010-0277(99)00012-8)

Wexler M, van Boxtel JJ. 2005. Depth perception by the active observer. *Trends in Cognitive Sciences* 9:431-438. DOI: [10.1016/j.tics.2005.06.018](https://doi.org/10.1016/j.tics.2005.06.018)

Wilson PN, Foreman N, Gillett R, Stanton D. 1997. Active versus passive processing of spatial information in a computer-simulated environment. *Ecological Psychology* 9:207-222. DOI: [10.1207/s15326969eco0903_3](https://doi.org/10.1207/s15326969eco0903_3)
